# Advances in Diagnosis and Treatment of HPV Ocular Surface Infections

**Published:** 2015

**Authors:** Chris D. Kalogeropoulos, Marilita M. Moschos

**Affiliations:** 1 Department of Ophthalmology, Ocular Inflammation Service, University of Ioannina, Greece; 2 Department of Ophthalmology, University of Athens, Greece

**Keywords:** HPV Ocular Surface Infections, Diagnosis, Treatment

More than 100 different mucosal types of human papillomavirus (HPV) have been identified. The existence of different HPV types at different sites of the human body was recognized in the late 1960s. Human papillomavirus infection is considered the most common sexually transmitted disease and can infect the ocular surface, as well (1-3). The mode of transmission of HPV infection to the conjunctiva in adults is considered autoinoculation from contaminated fingers in the majority of cases. 

Human papillomaviruses have been tangled in the pathogenesis and recurrence of conjunctival neoplasia, including conjunctival papillomas, conjunctival intraepithelial neoplasia (CIN) and even squamous cell carcinoma of the conjunctiva (SCCC). Human papillomavirus may coexist in SCCC lesions with other oncogenic viruses, such as the human immunodeficiency virus (HIV). According to their oncogenic potential, HPVs are divided into low - and high-risk types. The oncogenic properties of HPVs are attributed mainly to the viral oncoproteins E6 and E7. The involvement of HPV in the pathogenesis of pterygium remains controversial, although suggested by several studies using polymerase chain reaction (PCR) and immunohistochemical techniques.

Human papillomaviruses are DNA viruses that have a marked tropism for squamous epithelium explaining the association of HPV infection with squamous cell papilloma of the conjunctiva. On the other hand, the role of HPV infection in the etiology of SCCC remains unclear (1-4).

Human papillomaviruses types 6 and 11 are the most frequently found in conjunctival papillomas. Low-risk (LR) HPV 6 and HPV 11 are found in the majority of conjunctival papillomas along with dysplasia in several cases. In spite of such a dysplasia, carcinoma rarely develops in conjunctival papillomas. Other types found are HPV 33, HPV 45, and HPV 13. In addition, 6a and 45, two new subtypes, have been reported to be associated with conjunctival papilloma. On the other hand high-risk (HR) HPV 16 and HPV 18 have been also found in conjunctival papillomas; these types are strongly associated with the occurrence of high-grade uterine cervical intraepithelial neoplasia progressing to cervical cancer. However, according to the 2007 International Agency for Research on Cancer, available evidence on conjunctival carcinogenicity of HPV in humans is limited (4). 

Squamous cell carcinoma of the conjunctiva is a rare tumor that has been strongly linked with UV radiation and immunosuppression (particularly in HIV patients). Conjunctival intraepithelial neoplasia (CIN) is a precursor of SCCC but the role of HPV infection in the etiology of SCCC remains unclear. The DNA and mRNA of HPV 16 and HPV 18 corresponding to the E6 region have been detected in CIN. A few other studies have identified the presence of HPV 16 and HPV 18 in severe dysplastic lesions and carcinomas of the conjunctiva. HPV 6 and HPV 11 have also been found in severe dysplasias and carcinomas of the conjunctiva. In addition, mainly in HIV-positive patients, cutaneous HPV types (commonly HPV 5 and HPV 8) have been found in SCCC lesions. In contrast, a strong relationship of HPV and SCCC was not found in multiple other studies (4). 

Prevalence of conjunctival papillomas depends on geographical area, but generally is higher than that of conjunctival carcinomas. In some African countries with high HIV prevalence (i.e. Uganda, Tanzania) a possible role of both HPV and HIV as co-factors in SCCC pathogenesis has been suspected, even though controversial. It is noteworthy that even though there are no cross-sectional epidemiological studies, evidence suggests that people without explicit clinical presentation may host the virus and HPV DNA can be identified in an even asymptomatic conjunctiva. However, the worldwide dissemination of HPV infection deserves our attention for the successful management of ocular morbidity (5).

We have to bear in mind that, even though conjunctival papillomas are not life-threatening, they may be large enough to be displeasing or cosmetically unacceptable and affect vision. Furthermore, the recurrence rate for infectious papillomas is high; limbal papillomas have a recurrent rate of 40%. Therefore, accurate diagnosis is an indispensable step in preventing recurrences. The major clinical findings indicating conjunctival papilloma are papillomatous lesions (of exophytic growth pattern, sessile or pedunculated) characterized by reiterating fibro-vascular cores with a geometrically arranged set of red dots. Squamous cell papilloma with an infectious viral etiology has the tendency to recur after medical and surgical treatment. Most papillomas are benign but rarely they can undergo malignant transformation what is visible as inflammation, keratinization, or symblepharon formation (6).

The differential diagnosis of conjunctival papillomas includes a variety of tumors:

1) Benign tumors of the surface epithelium (i.e. keratoacanthoma and actinic keratosis)

2) Malignant lesions of the surface epithelium as CIN and SCCC,

3) Conjunctival lymphomas,

4) Vascular tumors (i.e. lymphangioma and capillary hemangioma),

5) Nonpigmented conjunctival melanomas, and

6) Secondary tumors.

There is a diffuse variant of conjunctival squamous cell neoplasia that can mimic chronic conjunctivitis and the differential diagnosis is difficult in cases of tumor thickening. Therefore, a conjunctival biopsy should be considered in cases of conjunctivitis lasting more than three months. The diagnosis of squamous conjunctival neoplasia is typically made by biopsy and invasion spreading into the substantia propria beneath the epithelium defines these lesions as carcinomas (2, 6). 

Tumor within the conjunctival epithelium does not have access to the lymphatic system (no metastatic potential). This tumor can extend onto the cornea (avascular and opaque in appearance), around the limbus but rarely inside the eye and orbit. Squamous conjunctival neoplasia commonly contains characteristic corkscrew-shaped blood vessels (6).

The medical history, slit-lamp examination, and the specific clinical and histopathological features of each tumor are used for accurate diagnosis. In the presence of a papillomatous growth pattern along with koilocytosis (nuclear pyknosis and cytoplasmic clearing), the morphological hallmark of HPV infection and mild epithelial dysplastic changes, further laboratory investigation to confirm the clinical diagnosis of HPV infection must be performed. Therefore, Immunohistochemical staining, in situ hybridization and PCR are the appropriate laboratory procedures in the detection of HPV and combination of the mentioned methods increases the diagnostic reliability (2, 5).

Immunohistochemical staining concerns the detection of HPV and p16 protein. The p16 INK4a (p16) is a cyclin-dependent kinase inhibitor and shows marked overexpression in cancerous and precancerous cervical lesions caused by persistent infections with HR HPV types. Immunohistochemical staining of the biopsy specimens is a natural crucial step regarding conventional histopathological findings in HPV infections. It is possible to directly isolate HPV DNA from a biopsy specimen with in situ hybridization (ISH). This method, whatsoever, needs a large quantity of purified DNA, and its sensitivity is relatively limited, especially in obtaining cells from the ocular surface via non invasive methodologies (for example exfoliation cytology techniques). In cases where the biopsy specimen is small with a limited quantity of HPV DNA, nucleic acid amplification assays can be used to increase the sensitivity and specificity of the test. Therefore, the material provided by invasive methods is preferred for laboratory examination of conjunctival lesions with suspected HPV infection (5).

Hybrid Capture II (HC-II) is a non radioactive signal amplification technique, accurate for mucosal lesions but is not appropriate for genotyping; it is useful in distinguishing HR from LR HPV types. Conversely, due to its high sensitivity, PCR is frequently associated with a high frequency of false-positive results. Southern blot, dot blot, reverse dot blot, digestion with restriction endonucleases or direct sequence analysis performed after DNA amplification can help increase the sensitivity and specificity of the test. More specifically real-time PCR or quantitative PCR (qPCR) permits rapid detection and quantification of the material during the various cycles of the PCR process (real time), considered the first choice assay for the detection of viral gene expression (5, 7).

However, the technique of sample collection, affecting the quantity of the HPV DNA of the isolated sample, and the use of various HPV DNA detection techniques with different sensitivity and specificity, are factors that may determine the detection rates of HPV infections. Combination of the described methods increases the rates of HPV detection. Nevertheless, it is imperative that a careful excision of the lesion and appropriate fixation of the specimen are preconditions for success of the diagnostic procedure. An excisional biopsy is preferred to an incisional biopsy whenever possible and consultation with a general pathologist or, ideally, an ophthalmic pathologist is compulsory. Performing an excisional biopsy is recommended to exclude premalignancy in adults (6).

Regarding squamous cell neoplasia, the lesion is removed surgically. After that, cryotherapy is applied to the adjacent conjunctiva and appears to be an effective technique, especially for squamous cell papillomas. Carbon dioxide (CO2) laser has also been used, what allows for precise tissue excision with minimal trauma and blood loss. Rapid healing occurs without significant scarring, edema, or symblepharon formation. Recurrence is low, resulting from the destruction of viral particles and papillomatous epithelial cells. Mitomycin-C (MMC) is an antineoplastic agent applied as 0.2 or 0.3 mg/mL dose via a cellulose sponge to the involved area(s) after surgical excision. The sponge is held in place for 3 minutes followed by meticulous irrigation. It is adjuvant to surgical removal but is also indicated for recalcitrant conjunctival papillomas or those refractive to previous multiple treatments; it is sometimes even administered to prevent recurrences of CIN and SCCC. In addition, amniotic membrane transplantation is used to restore extended conjunctival defects (2). In spite of MMC’s effectiveness and its setting up as a therapy asset, one should always keep in mind the potential of some rare complications such are symblepharon, corneal edema, corneal perforation, iritis, cataract, and glaucoma. Therefore, a close follow-up is recommended.

Beside surgical approach additional treatments concern (6):

1) Cimetidine (H2-receptor antagonist), indicated for recalcitrant and quite large conjunctival papillomas; cimetidine has been also found to enhance the immune system by inhibiting suppressor T-cell function and augmenting delayed-type hypersensitivity responses.

2) Interferon is an adjunct therapy to surgical excision of nonrecurring or recurrent multiple lesions. Alpha interferon is given intramuscularly for several months. Because of its antiviral and antiproliferative properties, this form of therapy is designed to suppress tumor cells. Additionally, topical interferon alpha-2b has been shown to be an effective adjunct therapy for small-to-medium size lesions but not for large lesions without surgical excision. Topical interferon alpha-2b can be utilized as an adjunctive therapy for recurring conjunctival papilloma and it is successful in treating also CIN lesions.

3) Dinitrochlorobenzene (DNCB), an immune modulator, may induce delayed hypersensitivity reaction causing the tumor to regress. The mechanism is not known. DNCB is applied directly to the papilloma once the patient has been sensitized to DNCB. This treatment modality is reserved for cases when surgical excision, cryoablation, and other treatment modalities fail (8).

Concerning squamous cell papillomas, the prognosis is generally good. However, recurrences of viral papilloma are not uncommon. On the other hand, recurrences of completely excised squamous cell papillomas are uncommon.

Surgical excision alone of CIN and SCCC lesions has been associated with frequent recurrences. This is because the tumor's edges and deep margins (often clear and avascular) are generraly difficult to determine, misleading the operator pretending the tumor is smaller than it is. Local freezing of the tumor nest (sclera and adjacent conjunctiva) has improved local management and decreased the incidence of tumor’s recurrence (2).

According to another approach, radiation therapy is administered to decrease tumor’s recurrence rate. In addition, topical chemotherapy, or "chemotherapy eye drops" have been found effective in several clinical trials. Therefore, large clinical trial is needed to compare the effectiveness of topical chemotherapy to excision, cryotherapy and combinations (6).

Intraocular penetration of tumor is extremely rare in developed societies; it is typically treated by enucleation of the eye or eye-wall resection. Orbital invasion brings along the risk of spread into the sinuses and brain, the most common cause of death related to this tumor. When squamous conjunctival neoplasia metastasizes outside of the eye and orbit, it can affect regional lymph nodes (preauricular, submandibular, and cervical) or/and the lungs and bone. In general, early detection allows for removal of these tumors with excellent local cure rates. Outdoor occupation, living close to the equator, tendency to sunburns and history of actinic skin lesions are risk factors. Theoretically, decreasing of sun exposure may prevent squamous cell lesions (6). 

As already mentioned, the role of HPV infection in human eye disease is controversial, but it is likely that HPV6/11 plays a role in the pathogenesis of conjunctival papilloma (2, 7). However, some cases illustrate the possible role of HPV in SCCC and the potentially devastating effects of this disease. The development of two vaccines, for prophylaxis from HPV infection concerning the types most commonly associated with anogenital cancers, has trigerred controversies with regard to the real benefit by a national immunization program to prevent cervical cancer. It seems that the greatest benefit in eye disease would be achieved administering the quadrivalent vaccine. The impact of the quadrivalent prophylactic vaccine against HPV types 6, 11, 16, and 18 on all HPV-associated genital disease was investigated in a population of sexually inexperienced (HPV-unexposed) women. Prophylactic vaccination was 95%–100% effective in reducing HPV 16 and 18 types related high-grade cervical, vulvar, and vaginal lesions and 97% effective in reducing HPV 6 and 11 types related genital warts (9).

Thus, in ophthalmology, the quadrivalent vaccine is expected to decrease the incidence of conjunctival papillomas due to HPV infection. In contrast, the HPV vaccination is not expected to prevent SCCC because HPV is not the main oncogenic agent in SCCC. Nevertheless, it will take many years before the benefits of a vaccination program become apparent, because even though papillomas occur in a relatively young, conjunctival carcinoma is usually a disease of the elderly (9).

 In addition, despite controversies in the medical literature concerning HPV involvement in pterygium development, the results of most studies agree that HPV is detected in at least a subgroup of pterygia. In these cases HPV infection may affect both pathogenesis and clinical behavior (including recurrence) of pterygium (10). Therefore, it would be interesting to explore the possibility of antiviral medications or even vaccination, which may represent novel options in the therapy of selected HPV-infected pterygia. 

However, clinicians should be aware of a possible bilateral uveitis and papillitis following HPV vaccination (11). Multiple transitory white dots of the retina as a manifestation of multiple evanescent white dot syndrome (MEWDS) have also been reported in the early period after HPV vaccination**.**

## DISCLOSURE

The authors report no conflicts of interest in this work.
